# The Paracrine Effect of Transplanted Human Amniotic Epithelial Cells on Ovarian Function Improvement in a Mouse Model of Chemotherapy-Induced Primary Ovarian Insufficiency

**DOI:** 10.1155/2016/4148923

**Published:** 2015-11-09

**Authors:** Xiaofen Yao, Yuna Guo, Qian Wang, Minhua Xu, Qiuwan Zhang, Ting Li, Dongmei Lai

**Affiliations:** The Center of Research Laboratory and Department of Gynecology, The International Peace Maternity and Child Health Hospital, School of Medicine, Shanghai Jiaotong University, Shanghai 200030, China

## Abstract

Human amnion epithelial cells (hAECs) transplantation via tail vein has been reported to rescue ovarian function in mice with chemotherapy-induced primary ovarian insufficiency (POI). To test whether intraperitoneally transplanted hAECs could induce therapeutic effect and to characterize the paracrine effect of transplanted hAECs, we utilized a chemotherapy induced mice model of POI and investigated the ability of hAECs and conditioned medium collected from cultured hAECs (hAECs-CM) to restore ovarian function. We found that transplantation of hAECs or hAECs-CM either 24 hours or 7 days after chemotherapy could increase follicle numbers and partly restore fertility. By PCR analysis of recipient mice ovaries, the presence of SRY gene was only detected in mice transplanted with male hAECs 24 hours following chemotherapy. Further, the gene expression level of VEGFR1 and VEGFR2 in the ovaries decreased, although VEGFA increased 2 weeks after chemotherapy. After treatment with hAECs or hAEC-CM, the expression of both VEGFR1 and VEGFR2 increased, consistent with the immunohistochemical analysis. In addition, both hAECs and hAECs-CM treatment enhanced angiogenesis in the ovaries. The results suggested that hAECs-CM, like hAECs, could partly restore ovarian function, and the therapeutic function of intraperitoneally transplanted hAECs was mainly induced by paracrine-mediated ovarian protection and angiogenesis.

## 1. Introduction

Primary ovarian insufficiency (POI), which used to be defined as premature ovarian failure or primary ovarian failure, is a subclass of ovarian dysfunction that has been caused by damage within the ovary [1]. POI is characterized by the triad of amenorrhoea, increased secretion of gonadotropins, and diminished production of estrogen under the age of 40 years. Chemotherapy for cancer has been suggested to be associated with POI. Ovarian tissue will often show follicle loss, cortical fibrosis, and vascular damage after chemotherapy by histologic examination [2–4]. Alkylating agents (such as cyclophosphamide) and busulfan appear to be high risk for inducing gonadotoxicity [5]. We used mice sterilized by intraperitoneal injection of busulphan and cyclophosphamide (Bu/Cy) to establish an infertile mice model [6].

Developed from the epiblast 8 days after fertilization and before gastrulation, human amniotic epithelial cells (hAECs) might maintain the plasticity of pregastrulation embryo cells. hAECs have been demonstrated to maintain the capability to differentiate into liver (endoderm), pancreas (endoderm), cardiomyocyte (mesoderm), and neural cells (ectoderm)* in vitro* [7]. Pluripotency, low immunogenicity, few ethical problems with usage, and nontumorigenicity make hAECs a useful source of stem cells for cell transplantation and regenerative medicine [8]. Transplanted hAECs have been shown to improve cardiac function in a rat model of myocardial infarction via injection of hAECs into the infarction area [9]. And intraperitoneal administration of hAECs into lung-injured mice decreased pulmonary fibrosis, reduced structural lung damage, and preserved lung function [10, 11]. Our previous study suggested that intravenously injected hAECs could transdifferentiate into granulose cells and restore folliculogenesis in a POI mouse model [12]. However, it is not clear whether hAECs could restore ovarian function through paracrine effects.

Vascular endothelial growth factor A (VEGFA) plays an important role in the regulation of angiogenesis in the ovary [13]. Although VEGFA is most well known as an angiogenic factor, it is also involved in many key events in the course of an ovulatory cycle, including follicular growth, ovulation, and corpus luteum development [14].* In vivo*, the number of primary and secondary follicles increased after injection of VEGF into rat ovaries [15]. In addition, both the antrum formation rate and the progression of meiosis to the MII stage were enhanced after adding VEGFA to the culture medium of caprine preantral follicles [16]. What is more, though inhibition of both VEGFR1 and VEGFR2 could reduce both vascular and follicle development, inhibition of VEGFR2 blocks follicle progression but does not necessarily disrupt vascular development in perinatal rat ovaries, which suggests that VEGFA and its receptors could potentially be involved in nonangiogenic and angiogenic mechanisms in the regulation of follicle development [17].

Herein we investigated if hAECs transplantation could restore Bu/Cy-damaged ovarian function by intraperitoneal administration and whether the therapeutic efficacy is mediated by the paracrine effect. We also detected the expression of VEGFA and its receptors in the mice ovaries induced by Bu/Cy administration and analyzed the effects of transplanted hAECs and factors secreted by hAECs on the ovarian angiogenesis.

## 2. Materials and Methods

### 2.1. Isolation and Culture of hAECs

Male hAECs were isolated and cultured as described previously [12]. In brief, discarded placentas from male fetus were obtained at term pregnancy during uncomplicated caesarean sections with written informed consent from woman who tested negative for HIV-I and hepatitis B and C. The indication for caesarean section is breech presentation, repeat operation, fetal distress, and twins. The institutional ethics committee approved the use of human amnions for this project.

After mechanically peeled from the chorionic portion of the placenta, the amniotic membrane, which looks like a translucent sheet, was placed in 250 mL flasks containing Dulbecco's modified eagle medium (DMEM)/F12. After cut with a razor to yield 0.5 to 1.0 cm^2^ segments, the placental segments were digested with 0.25% trypsin/EDTA at 37°C for 45 min. Centrifuged at 250 ×g for 5 minutes at 25°C, the cells were resuspended with PBS after the supernatant was discarded. Then after being filtered through a 100 *μ*m cell strainer, washed with PBS once, and resuspended, the resulting cell suspensions were seeded in a six-well plate in DMEM/F12 medium supplemented with 10% fetal bovine serum (FBS, Gibco, Grand Island, NY, USA), streptomycin (100 U/mL; Gibco), penicillin (100 U/mL; Gibco), and glutamine (0.3 mg/mL; Gibco) and incubated at 37°C 5% CO_2_ in humidified air. Once hAECs reached 80 to 90% confluence, cells were ready for experiments.

### 2.2. Immunofluorescence Staining

hAECs were fixed with 4% paraformaldehyde for 20 minutes at room temperature and then washed twice (5 minutes each) with PBS. Cells were permeabilized with 0.1% Triton X-100 for 10 minutes at room temperature and then washed twice with 1 × PBS. Then cells were blocked with blocking solution for 30 minutes and incubated overnight at 4°C with anti-Cytokeratin 18 (mouse anti-human 1 : 200, Boster, Wuhan, China), anti-CD34 antibody (rabbit anti-human 1 : 100, Abcam, Cambridge, MA, USA), and anti-Vimentin (rabbit anti-human 1 : 100, Cell Signaling Technology, Danvers, MA, USA). Washed three times with PBS, the cells were probed with FITC-labeled IgG (1 : 200, Santa Cruz, CA, USA) or Rhodamine- (TRITC-) labeled IgG (1 : 100, Invitrogen, CA, USA). Fluorescence images were obtained with a Leica DMI3000 microscope (Heidelberg, Germany).

### 2.3. Preparation of Conditioned Medium (CM) from Cultured hAECs

The CM of hAECs was prepared according to a protocol described previously by Yang et al. [18], with minor modifications. Eighty percent of confluent hAECs were washed with PBS twice and then fed with serum-free medium for 24 hours. The medium was then collected and used for* in vivo* experiments. For each animal, we used CM generated by 4 × 10^6^ hAECs. The CM was centrifuged at 300 g for 5 minutes and sterilized through a 220 nm filter. The collected CM was concentrated using Amicon Ultra-15 centrifugal filter units (3 kDa cut-off; Millipore).

### 2.4. Animals

Six-week-old female C57BL/6 wild-type mice were purchased from Shanghai SLAC Laboratory Animal Co., Ltd. All animals were maintained on 12-hour light/dark cycles with food and water available ad libitum. To establish the POI model of chemotherapy-induced ovarian damage, a total of 85 mice used as recipients were sterilized by one intraperitoneal (IP) injection of Bu/Cy (busulphan, 30 mg/kg, and cyclophosphamide, 120 mg/kg, both resuspended in DMSO) [6]. All procedures involving animals were approved by the Institutional Animal Care and Use Committee of Shanghai and were conducted in accordance with the National Research Council Guide for Care and Use of Laboratory Animals. After injection, Bu/Cy-treated and control animals were weighed twice a week at 9 a.m.

### 2.5. IP Injection of hAECs and hAECs-CM

The C57BL/6 wild-type mice were randomly divided into six groups. The normal control mice received no treatment (*n* = 20). In the Bu/Cy group, the mice were administered Bu/Cy only (*n* = 15). In the Bu/Cy + hAECs (24 h) group (*n* = 20), mice received Bu/Cy administration and then an IP transplantation of 4 × 10^6^ hAECs in a volume of 0.2 mL PBS 24 h later. In the Bu/Cy + hAECs-CM (24 h) group (*n* = 15), mice received Bu/Cy administration and then an IP injection of 0.2 mL CM generated by 4 × 10^6^ hAECs 24 h later. In the Bu/Cy + hAECs (7 d) group (*n* = 20), mice received Bu/Cy administration and then an IP transplantation of 4 × 10^6^ hAECs 7 days later. In the Bu/Cy + hAECs-CM (7 d) group (*n* = 15), mice received Bu/Cy administration and then an IP injection of CM generated by 4 × 10^6^ hAECs 7 days later. The administration of hAECs or hAECs-CM was repeated the following day. While five mice in each group were kept for mating experiments, the other mice were culled either 14 or 28 days following Bu/Cy administration or the second treatment. The bilateral ovaries were collected from each animal for analysis.

### 2.6. Mating Experiment

The control mice, Bu/Cy-treated only mice, and mice treated with Bu/Cy and then hAECs or hAECs-CM were housed with C57BL/6 male mice one month after chemotherapy. Adult males of proven fertility were housed with females at a ratio of 1 : 2. The number of litters per pregnancy was recorded.

### 2.7. Ovarian Follicle Counts and Morphologic Analysis

One month after treatment, the ovaries were collected and the follicles were detected and classified. The removed ovaries were fixed in 4% paraformaldehyde for at least 24 hours. After fixation, the ovaries were dehydrated, paraffin-embedded, serially sectioned at 5 *μ*m, and mounted on glass microscope slides. Routine hematoxylin and eosin (H&E) staining was performed for histologic examination with light microscopy. Primordial, primary, secondary, and antral follicles were counted in every fifth section. Only follicles containing an oocyte were counted to avoid counting any follicle twice. Follicles were classified as follows: primordial follicle, oocyte surrounded by a single layer of squamous granulosa cells; primary follicle, intact enlarged oocyte with a visible nucleus and one layer of cuboidal granulosa cells; secondary follicle, more than one layer of cuboidal granulosa cells without antral space; antral follicles (including preovulatory follicles), emerging antral spaces [19].

### 2.8. Real-Time Quantitative PCR

The hAECs cells were collected when the cells reached 80 to 90% confluence. And ovarian samples were collected 2 weeks after last treatment. Then total RNA was isolated from the ovarian samples and 500 ng of total RNA from each sample was reverse transcribed using the primescript RT reagent kit (Takara Bio Inc., Shiga, Japan). Real-time PCR was performed on cDNA using SYBR Premix Ex Taq (Takara) on the Mastercycler ep realplex (Eppendorf, Hamburg, Germany). All reactions were carried out in triplicate, using a 25 *μ*L volume. In brief, PCR amplification was carried out using an initial denaturation at 95°C for 5 min, followed by 40 cycles for 30 sec at 95°C, 30 sec at 60°C, and 30 sec at 72°C. A sample lacking template DNA was used as a negative control. The primers for the genes are provided in Tables 1 and 2.

### 2.9. Immunohistochemical Analysis

Ovaries from treated and control animals were fixed, dehydrated, vitrified, and embedded in paraffin. The ovarian sections (5 *μ*m thick) were deparaffinized with xylene and hydrated using an ethanol gradient. The hydrated sections were washed in 3% hydrogen peroxide in methanol for 20 min at room temperature and blocked in PBS (Sigma) containing 3% BSA (Sigma) for 30 min. Sections were treated with primary antibodies including rabbit anti-CD34 antibody (1 : 200; Abcam, Cambridge, MA, USA), rabbit anti-VEGFA antibody (1 : 50; Abcam), rabbit anti-VEGFR1 antibody (1 : 200; Cell Signaling Technology, Danvers, MA, USA), or rabbit anti-VEGFR2 antibody (1 : 200; Abcam) for antibody detection at 4°C overnight. After washing, slides were then incubated with HRP labeled anti-rabbit antibody (Peroxidase reaction kits, Vector Laboratories, Burlingame, CA, USA). Peroxidase substrate was developed by using a DAB (3,39-diaminobenzidine) substrate kit (Vector Laboratories). Slides were counterstained with hematoxylin QS (Vector Laboratories) and were dehydrated and mounted with VectaMount Permanent Mounting Medium (Vector Laboratories).

### 2.10. Determination of Microvessel Density (MVD)

Angiogenesis was measured by MVD, which was assessed by light microscopic analysis (400×) for areas of the sections containing the most microvessels. Two independent researchers who were blinded to the experimental conditions were assigned to observe and calculate the MVD in five sections at ten sections intervals. Microvessel was defined as a brown-staining endothelial cell cluster with incomplete CD34+ endothelial cell–cell contact. The microvessel numbers from the five sections were counted and calculated for the mean number of microvessels per ovary [20]. Further, sections from five ovaries of each group were investigated for the average MVD.

### 2.11. PCR Analysis

To predict whether hAECs from male fetus placentas could migrate to the mouse ovaries, a multiplex PCR for amplification of the Y-specific* SRY* sequences was done as suggested by Tungwiwat et al. [21], with little modification. Ovarian DNA was extracted using the TIANamp Genomic DNA Kit (TIANGEN, China) with the protocol supplied by the manufacturer. The* SRY*-specific sequence was amplified firstly by PCR primer pair, Y1.5 (5′-CTAGACCGCAGAGGCGCCATC-3′) and Y1.6 (5′-TAGTACCCACGCCTGCTCCGG-3′). Using the first PCR product as a template, the SRY-specific sequence was amplified by another primer pair Y1.7 (5′-CATCCAGAGCGTCCCTGGCTT-3′) and Y1.8 (5′-CTTTCCACAGCCACATTTGTC-3′). Ten microliters of each product was analyzed on a 2% agarose gel electrophoresis and visualized under UV light after ethidium bromide staining. The nested Y-specific fragment* SRY* gene was 198 bp in length [21]. Identifications of the Y-specific sequences in hAECs from male and female fetus placentas were used as positive and negative controls. Ovarian DNA of female mice without hAECs injection was also used as template for negative control.

### 2.12. Data Analysis and Statistics

Data were expressed in each experimental group as mean ± SEM. Statistical significance was determined by statistical analysis software (GraphPad Prism, GraphPad Software Inc., USA) and evaluated with Student's* t*-test. Statistical significance was accorded when *P* < 0.05.

## 3. Results

### 3.1. Characterization of Isolated hAECs

**Figure 1:**
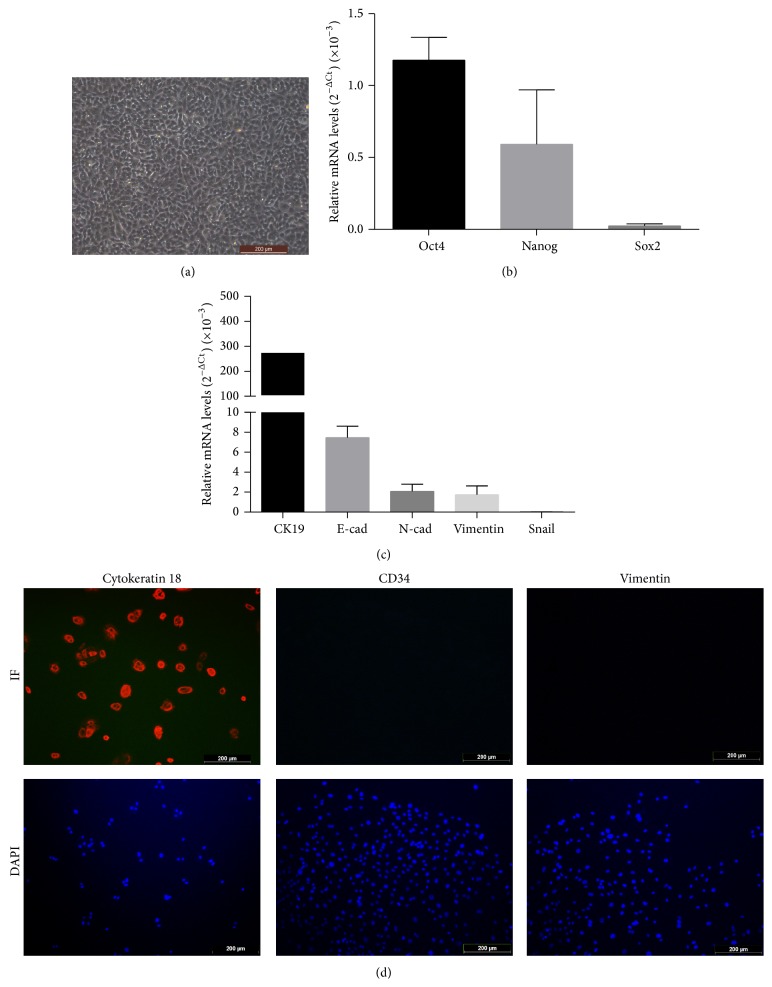
Identification and characterization of isolated hAECs. (a) The representative characteristics of hAECs under light microscope. (b) Expression of* Oct-4, Nanog, *and* Sox2* (relative to a beta-actin internal control) in cultured hAECs by real-time PCR analysis. The results presented were the average values from five different donors. (c) The analysis of epithelial and mesenchymal markers expression (relative to a beta-actin internal control) in isolated hAECs by real-time PCR. The results presented were the average values from five different donors. (d) Immunofluorescence detection of Cytokeratin 18, CD34, and Vimentin in cultured hAECs. Scale bar: 200 *μ*m.

Under light microscope, the hAECs formed confluent cobblestone-shaped monolayer epithelial cells (Figure 1(a)). To examine the stem cell specific genes and epithelial gene in hAECs, real-time PCR was done. Consistent with previous studies [7], we found that the isolated AE cells expressed* Oct-4, Nanog, *and* Sox2*, which are all involved in cellular pluripotency [22–24]. In addition, we detected the expression of epithelial (*Cytokeratin 19* (*CK19*) and* E-cadherin*) and mesenchymal (*N-cadherin, Vimentin, *and* Snail*) markers in cultured cells. As shown in Figure 1(c), both* E-cadherin *and* Cytokeratin 19* were highly expressed in cultured hAECs cells. Comparatively, mRNA expression of mesenchymal cell marker, including* N-cadherin* and* Vimentin,* was low and the expression of* Snail *gene was scarcely detected.

To further identify the purity of the freshly isolated hAECs, immunofluorescence staining was done. The cells were clarified with positive staining against Cytokeratin 18 and negative for CD34 and Vimentin (Figure 1(d)). Almost all freshly isolated hAECs were Cytokeratin positive. Simultaneously, no CD34 staining was observed, which indicated that the isolates are not contaminated with hematopoietic stem cells such as umbilical cord blood or embryonic fibroblasts. Meanwhile, absence of Vimentin suggested that the hAECs were not contaminated with mesenchymal cells from amniotic membrane.

### 3.2. Effect of hAECs and hAECs-CM Treatment after Bu/Cy Administration on Body Weight

**Figure 2:**
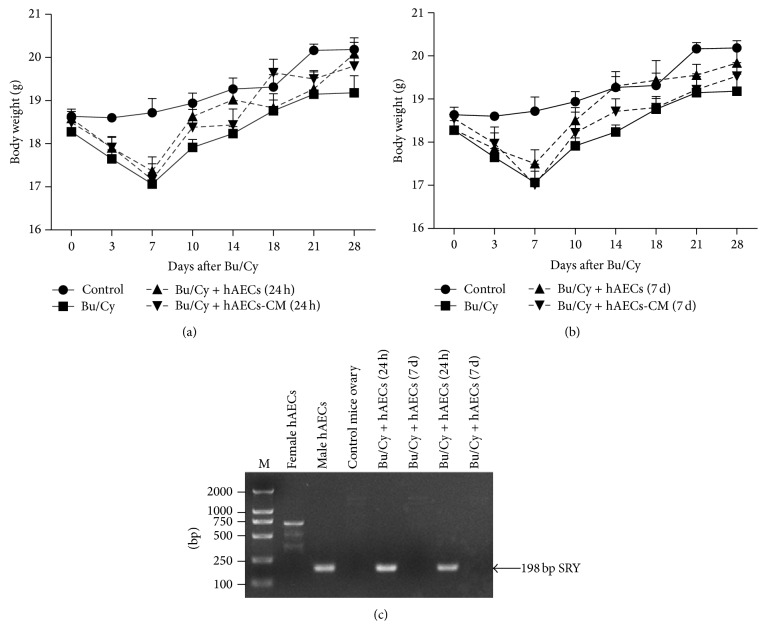
The effect of human hAECs and hAECs-CM administration on mice body weight and the detection of transplanted hAECs. (a) Weight of mice treated with hAECs and hAECs-CM 24 hours after chemotherapy. The weight of Bu/Cy-treated administrated mice decreased significantly 7 days after chemotherapy when compared with mice in the control group (*P* < 0.01). Mice of hAECs- and hAECs-CM-treated groups weighed more compared with Bu/Cy mice treated with Bu/Cy administrated at any time point, although the differences were not significant. (b) The weight of mice that received Bu/Cy treatment and then an IP injection of hAECs or hAECs-CM 7 days later. (c) A representative gel electrophoresis of detection of the* SRY* gene in female mice ovaries treated with male hAECs by a simultaneous nested PCR analysis. The nested amplified product of the* SRY* sequence on the Y chromosome is 198 bp in length.

To assess the effect of human hAECs and hAECs-CM on total body weight, wild-type female mice were sterilized by pretreatment with Bu/Cy and then intraperitoneally administrated with hAECs or hAECs-CM. Compared to normal control mice, administration of Bu/Cy resulted in a significant reduction in body weight over 7 days (18.72 ± 0.33 g versus 17.07 ± 0.26 g, *P* < 0.01, Figures 2(a) and 2(b)). No significant difference was observed between Bu/Cy-treated mice and mice that received hAECs or hAECs-CM treatment after Bu/Cy administration at any time point.

### 3.3. hAECs Transplanted 24 h after Bu/Cy Administration Can Infiltrate into the Damaged Ovarian Tissues

To confirm whether male hAECs could transfer into recipient ovaries, PCR analysis of the* SRY* sequences on the Y chromosome was done. As shown in Figure 2(c), genomic DNA prepared from male hAECs clearly demonstrated the presence of 198 bp* SRY*-specific sequences. Genomic DNA from both female hAECs and C57BL/6 mouse ovaries was used as negative control, with some nonspecific bands and absence of the 198 bp* SRY*-specific sequences. Interestingly, while the 198 bp fragments were identified on the ovarian samples of mice treated with male hAECs 24 hours after chemotherapy, there was no detectable product on the ovarian samples from mice treated with male hAECs 7 days after chemotherapy.

### 3.4. Both hAECs Transplantation and hAECs-CM Treatment Increase Follicle Number at Varied Stages in POI Mice

**Figure 3:**
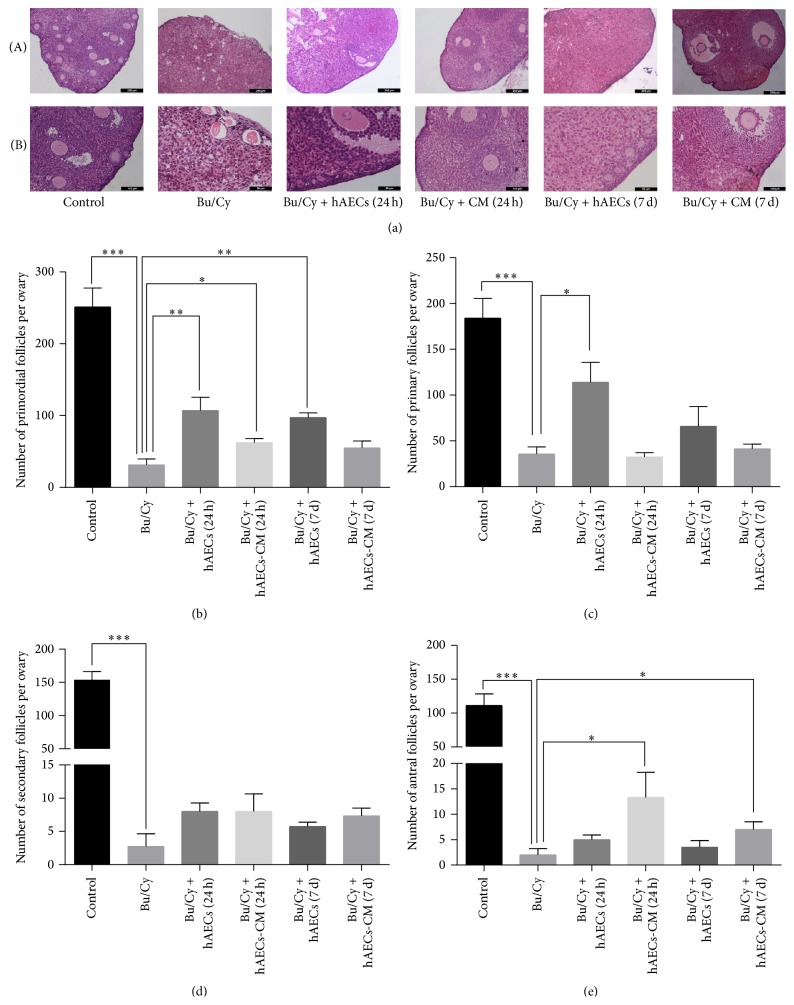
Histological analysis of mice ovaries and the follicle number count after treatment with hAECs or hAECs-CM. (a) Ovary tissues from each group were stained with hematoxylin and eosin. Numbers of oocyte-containing follicles at all stages were classified and counted in every fifth section. The primordial follicles (b), primary follicles (c), secondary follicles (d), and antral follicles (e) were identified and calculated. Data are shown as mean ± SEM, ^*∗*^ *P* < 0.05, ^*∗∗*^ *P* < 0.01, ^*∗∗∗*^ *P* < 0.001. Scale bar: 200 *μ*m (Panels (a)(A)) and 50 *μ*m (Panels (a)(B)), respectively.

To determine whether hAECs transplantation and hAECs-CM treatment via peritoneal cavity could restore ovarian folliculogenesis, the serial sections obtained from ovaries 1 month after hAECs and hAECs-CM treatment were stained with H&E. As shown in Figure 3(a), administration with Bu/Cy induced follicle loss, interstitial fibrosis, and atretic follicles. However, histological evaluations revealed that hAECs or hAECs-CM treatment after chemotherapy increased follicle number in recipient ovaries.

To further calculate the number of follicles in all stages, every fifth section was analyzed, and the number of primordial, primary, secondary, and antral follicles was counted using light microscopy. Compared with normal control group, the number of primordial (251.25 ± 26.35 versus 31.25 ± 8.5, *P* < 0.001), primary (184 ± 21.57 versus 35.75 ± 7.7, *P* < 0.001), secondary (153.5 ± 12.77 versus 2.75 ± 1.89, *P* < 0.0001), and antral follicles (111.25 ± 17.28 versus 2 ± 1.22, *P* < 0.001) decreased significantly in Bu/Cy-treated mice. In the ovaries of mice treated with hAECs 24 hours after chemotherapy, the number of primordial (107 ± 18.57) and primary follicles (114 ± 21.81) increased significantly compared to those in the Bu/Cy-treated group (*P* < 0.05, Figures 3(b) and 3(c)). However, there is no significant difference in the amounts of secondary and antral follicles between the hAECs-treated group and the Bu/Cy-treated group. In addition, the primordial follicles of mice treated with hAECs-CM 24 hours after Bu/Cy administration and mice transplanted with hAECs 7 days after Bu/Cy administration increased significantly (62.33 ± 5.67 and 97.33 ± 6.44, resp.). Moreover, compared with Bu/Cy-treated mice, the antral follicles of the hAECs-CM-treated group increased significantly, whether they were treated 24 hours or 7 days after chemotherapy (13.33 ± 4.91 versus 2 ± 1.22 and 7 ± 1.52 versus 2 ± 1.22, resp., *P* < 0.05, Figure 3(e)).

### 3.5. hAECs Transplantation and hAECs-CM Treatment Improve the Fertility of POI Mice

**Figure 4:**
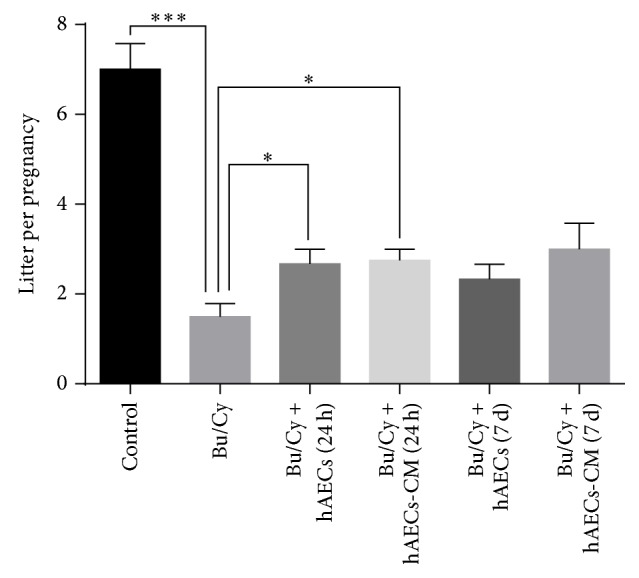
Intraperitoneal injection of hAECs and hAECs-CM could partly restore fertility in mice treated with chemotherapy. The litter size per pregnancy was recorded. Data represent means ± SEM, ^*∗*^ *P* < 0.05, ^*∗∗∗*^ *P* < 0.001.

To estimate whether the treatment of hAECs or hAECs-CM via peritoneal cavity could restore the ovarian function, the female mice were naturally mated with male mice of proven fertility four weeks after treatment. The number of litters per pregnancy was calculated. The average number of litters in the group of Bu/Cy-treated mice (1.5 ± 0.29) was significantly lower than that in the normal control group (7 ± 0.58). Treatment of hAECs and hAECs-CM 24 hours after Bu/Cy administration significantly increased the litters per pregnancy (2.7 ± 0.3 and 2.75 ± 0.25, resp.) compared with Bu/Cy-treated mice. The litters per pregnancy in mice treated with hAECs or hAECs-CM 7 days after Bu/Cy administration increased, but there were no significant differences. Therefore, mice fertility can be partly improved by injecting hAECs and hAECs-CM into the abdominal cavity of Bu/Cy-treated mice (Figure 4).

### 3.6. The VEGFA Pathway Is Involved in the Therapeutic Efficacy of hAECs and hAECs-CM

**Figure 5:**
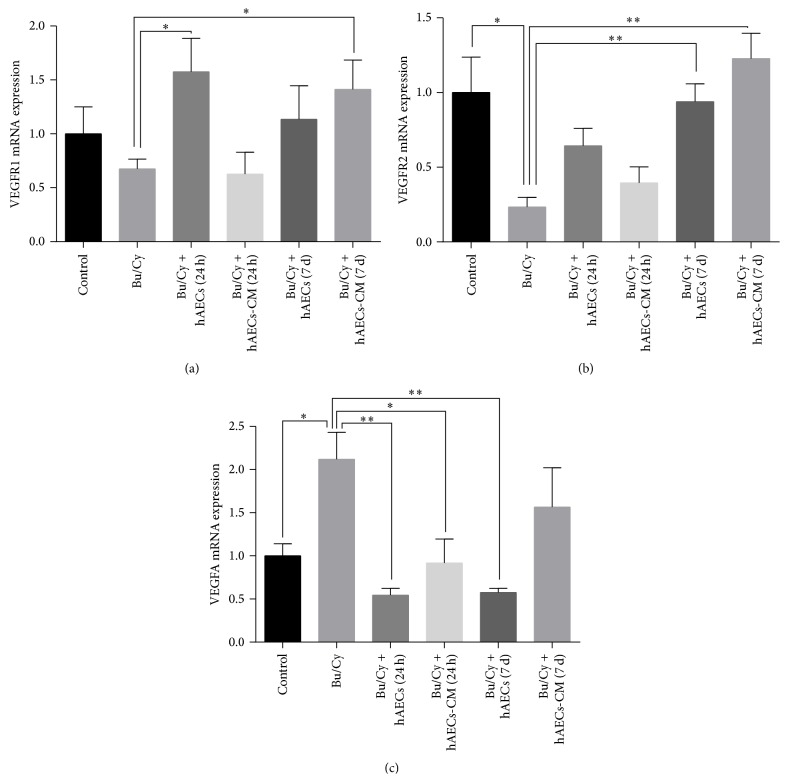
Real-time PCR analysis for VEGFR1, VEGFR2, and VEGFA expression in mice ovaries of different groups 2 weeks after treatment. ^*∗*^ *P* < 0.05, ^*∗∗*^ *P* < 0.01.

As it has been reported that VEGFA and its receptors could potentially be involved in the regulation of follicle development [17], we attempted to examine whether VEGFA pathway molecules are involved in ovarian restoration after treatment with hAECs and hAECs-CM by analyzing the RNA and protein expression levels using real-time PCR and immunohistochemistry. As shown in Figures 5(b) and 5(c), the VEGFR2 expression level significantly decreased in the ovaries 2 weeks after chemotherapy (*P* < 0.05), along with increased VEGFA expression (*P* < 0.05) in mRNA levels compared with the normal control group. After treatment with hAECs or hAECs-CM, both VEGFR1 and VEGFR2 were higher expressed than Bu/Cy-treated only mice (Figures 5(a) and 5(b)).

**Figure 6:**
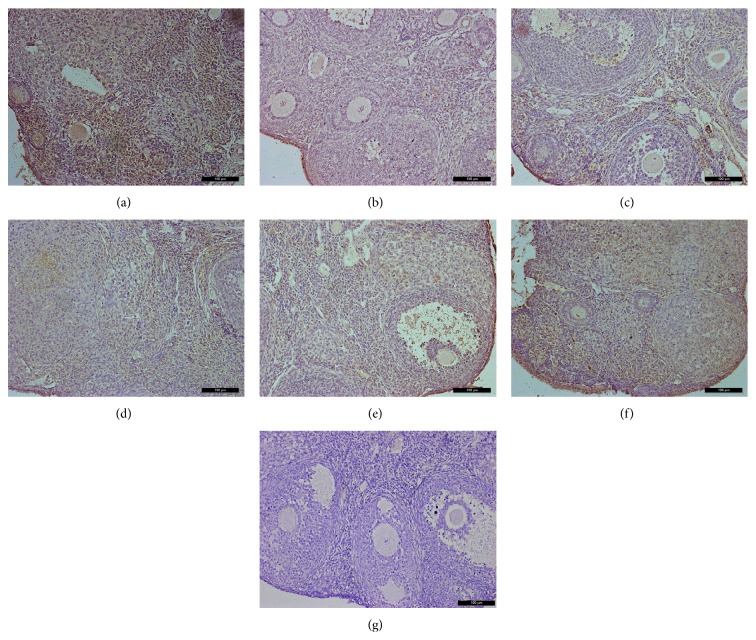
Immunohistochemical analysis for VEGFR1 expression in mice ovaries of different groups 2 weeks after treatment; ovarian sections with no primary antibody were served as negative controls. (a) control group; (b) Bu/Cy-treated group; (c) Bu/Cy + hAECs (24 h) group; (d) Bu/Cy + hAECs-CM (24 h) group; (e) Bu/Cy + hAECs (7 d) group; (f) Bu/Cy + hAECs-CM (7 d) group; (g) negative control. Scale bar: 100 *μ*m.

**Figure 7:**
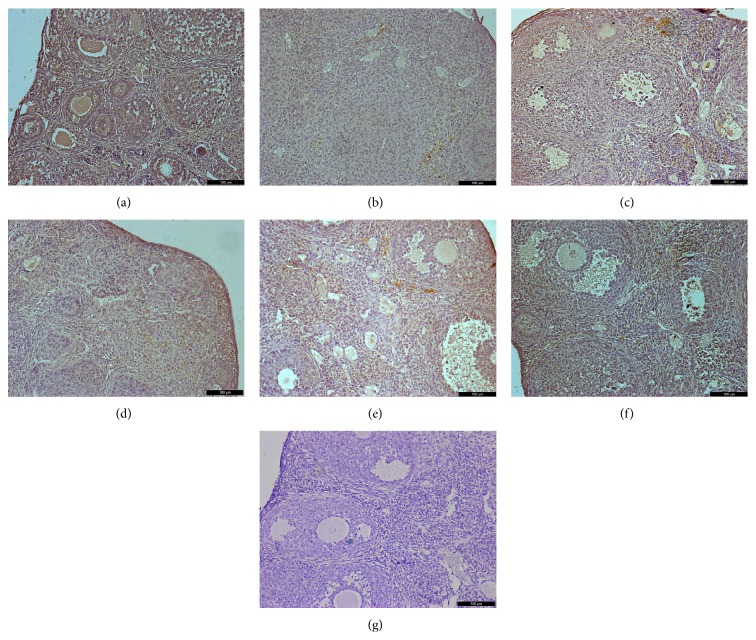
Immunohistochemical analysis for VEGFR2 expression in mice ovaries of different groups 2 weeks after treatment; ovarian sections with no primary antibody were served as negative controls. (a) control group; (b) Bu/Cy administrated group; (c) Bu/Cy + hAECs (24 h) group; (d) Bu/Cy + hAECs-CM (24 h) group; (e) Bu/Cy + hAECs (7 d) group; (f) Bu/Cy + hAECs-CM (7 d) group; (g) negative control. Scale bar: 100 *μ*m.

**Figure 8:**
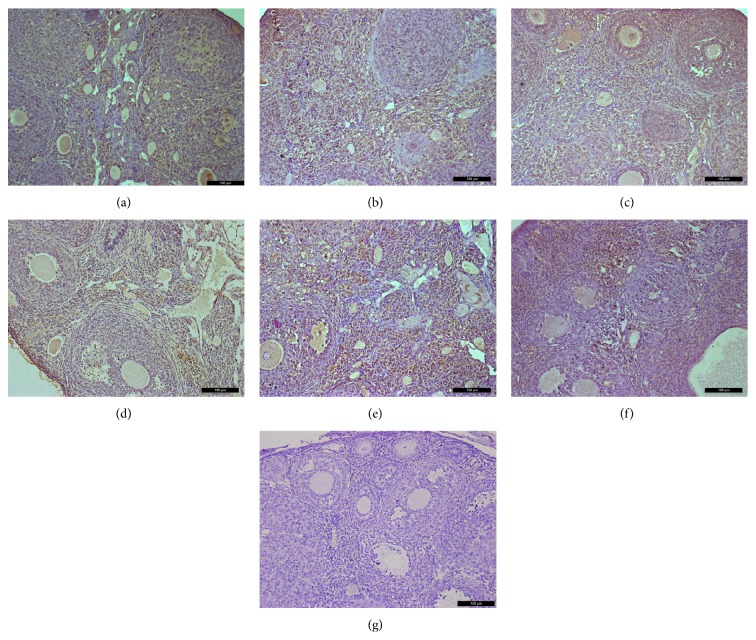
Immunohistochemical analysis for VEGFA expression in mice ovaries of different groups 2 weeks after treatment; ovarian sections with no primary antibody were served as negative controls. (a) control group; (b) Bu/Cy administrated group; (c) Bu/Cy + hAECs (24 h) group; (d) Bu/Cy + hAECs-CM (24 h) group; (e) Bu/Cy + hAECs (7 d) group; (f) Bu/Cy + hAECs-CM (7 d) group; (g) negative control. Scale bar: 100 *μ*m.

Immunohistochemical analysis of ovaries 2 weeks after treatment showed that VEGFA and its two receptors had similar expression at the protein level as that of mRNA expression levels (Figures 6–8). Immunohistochemical studies revealed that VEGFA, VEGFR1, and VEGFR2 were frequently observed in the normal control group both at 2 weeks and at 1 month. Interestingly, decreased expression of VEGFA, VEGFR1, and VEGFR2 was observed 1 month after chemotherapy in the Bu/Cy-treated group. However, the expression of these proteins in hAECs- or hAECs-CM-treated mice ovaries was partly restored to the levels of those seen in control group (see Supplemental Figures 1–3 in Supplementary Material available online at http://dx.doi.org/10.1155/2016/4148923).

### 3.7. hAECs Transplantation and hAECs-CM Treatment Increase the MVD in Ovarian Tissues of POI Mice

**Figure 9:**
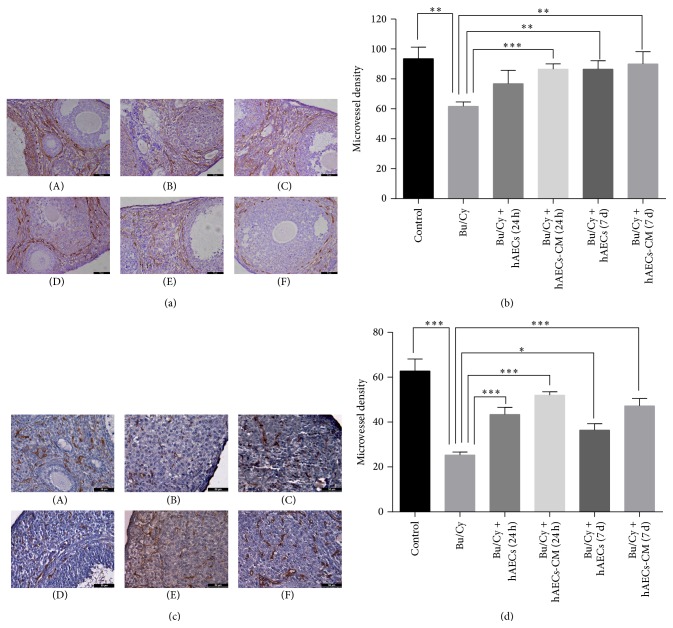
Angiogenesis on Bu/Cy-treated ovaries after intraperitoneal injection of hAECs or hAECs-CM. Immunohistochemistry for CD34 ((a) and (c)) and microvessel density ((b) and (d)) were examined on ovaries obtained 2 weeks ((a) and (b)) or 1 month after treatment ((c) and (d)). Data were given as mean ± SEM. (A) control group; (B) Bu/Cy administrated group; (C) Bu/Cy + hAECs (24 h) group; (D) Bu/Cy + hAECs-CM (24 h) group; (E) Bu/Cy + hAECs (7 d) group; (F) Bu/Cy + hAECs-CM (7 d) group. ^*∗*^ *P* < 0.05, ^*∗∗*^ *P* < 0.01, ^*∗∗∗*^ *P* < 0.001. Scale bar: 100 *μ*m.

CD34, an endothelial marker used in this study for vessel evaluation, was stained by immunohistochemistry in ovarian tissues to evaluate the MVD. Histological evaluations demonstrated that the number of CD34-staining vessels was significantly decreased in ovarian tissues after Bu/Cy administration (Figures 9(a) and 9(c)). As shown in Figures 9(b) and 9(d), compared with normal control group, ovarian MVD was diminished by 34% (*P* < 0.01) and 60% (*P* < 0.001), respectively, in mice ovaries 2 weeks and 1 month after Bu/Cy administration. One month after treatment with hAECs or hAECs-CM, CD34 was significantly higher in all four treatment groups than the Bu/Cy-treated group, regardless of the timing of hAECs or hAECs-CM administration. There were no significant differences among the four treatment groups 2 weeks after treatment (Figures 9(a) and 9(b)). However, 1 month after treatment, levels of ovarian MVD in the hAECs-CM-treated group were significantly higher than in the hAECs-treated group regardless of time of injection, either 24 hours or 7 days after chemotherapy (*P* < 0.05, Figures 9(c) and 9(d)).

## 4. Discussion

hAECs have attracted interest for their possible use for regenerative medicine because of their pluripotency, low immunogenicity, nontumorigenicity, and few ethical problems with their usage [8]. There are two possible mechanisms to explain the therapeutic efficiency of hAECs transplantation. One is the differentiation potential of the transplanted cells to damaged cells. The other is the ability to secrete functional or protective factors from the transplanted cells. These factors may stimulate proliferation, inhibit apoptosis of cells residing in the damaged organs, and promote angiogenesis of damaged tissues to improve oxygen delivery and metabolic exchange [25, 26]. To address the mechanism underlying the beneficial effect of hAECs in ovarian restoration, we used a preclinical mouse model of Bu/Cy-induced ovarian failure. Then we injected hAECs and hAECs-CM into the peritoneal cavity of the Bu/Cy-treated mice to investigate whether hAECs could migrate to the ovary to revive ovarian function and whether factors secreted by hAECs could also induce similar therapeutic effect.

In 2002, Wang and colleagues [27] reported that real-time PCR was sensitive, species- and sex-specific in detecting the male* SRY* gene after sex-mismatched liver cell transplantation. More recently, PCR detection of the* SRY* gene in the ovaries was suggested as an effective method to investigate whether male marrow-derived mesenchymal stem cells could immigrate into chemotherapy-damaged ovaries to restore ovarian function [3, 28]. Interestingly, in our study, cell tracking experiments using PCR analysis confirmed the presence of donor male hAECs-derived* SRY* gene in ovaries after hAECs were transplanted 24 hours after chemotherapy. However, mice treated with hAECs 7 days after chemotherapy did not have* SRY* gene in any ovary. It was reported that women exposed to a variety of chemotherapy regimes could induce subcapsular focal cortical fibrosis [4], which may inhibit hAECs from immigrating into ovaries 7 days after chemotherapy. In addition, a maximal benefit was achieved when hAECs were transplanted 24 hours (versus 7 days) after Bu/Cy administration. The number of primordial, primary, secondary, and antral follicles was higher in mice transplanted with hAECs 24 hours after chemotherapy than after 7 days, though the difference was not significant. These results suggest that the timing of the transplantation was important and whether hAECs could migrate to the ovary or not could affect the therapeutic effect.

Recently, more and more studies had employed factors released from cells as cellular therapeutics in regenerative medicine. It was demonstrated that conditioned media isolated from adipose-derived stem cells (ADSC) had a similar protective effect as intact ADSC in improving both cardiac function of infarcted hearts and lung vascular protective function after lung tissue microvascular injury [18, 29]. These results strongly support major involvement of paracrine effects in regenerative medicine. Similar to these results, IP injection of hAECs-CM in our study partly restored follicle number and improved fertility when compared with Bu/Cy-treated mice. Though treatment 24 hours after Bu/Cy treatment seemed more effective than treatment after 7 days, there is no significant difference. To our surprise, hAECs-CM injection significantly increased the number of antral follicles regardless of the timing of administration. In brief, IP injection of 0.2 mL concentrated hAECs-CM collected from 4 × 10^6^ hAECs (30-fold concentrated) seemed sufficient to improve ovarian function of chemotherapy-induced ovarian damage. Hence, hAECs-CM might be a meaningful treatment strategy for clinical application.

It was reported that VEGF is a factor that acts by stimulating the mitosis of endothelial cells and by increasing vascular permeability [30, 31], which may result in the accumulation of antral fluid, formation of antrum in the growing follicles, and, finally, inducement of follicle rupture [14, 32, 33]. Actually, addition of VEGF to the culture medium improved the development of caprine preantral follicles cultured* in vitro*, allowing the production of mature oocytes [16]. Therefore, the therapeutic effect of hAECs may partly result from hAEC-derived paracrine factors, especially VEGFA.

In the present study, we analysed the expression of VEGFA and its receptors (VEGFR1 and VEGFR2) in the ovaries by real-time PCR and immunohistochemical analysis. Compared with normal control mice, the expression of VEGFR1 and VEGFR2 was decreased 2 weeks after Bu/Cy administration, along with increased VEGFA expression. IP transplantation of hAECs or hAECs-CM into the Bu/Cy-treated mice induced the expression of VEGFR1 and VEGFR2 2 weeks after treatment. We speculated that the increased expression of VEGFA in Bu/Cy-treated mice was transient and caused by autoregulation as a response to the reduction of VEGFR1 and VEGFR2 expression. Actually, the expression of VEGFA, VEGFR1, and VEGFR2 was decreased 1 month after chemotherapy. In addition, the transplantation of hAECs or hAECs-CM into the ovaries that showed decreased angiogenesis due to Bu/Cy treatment resulted in the induction of more CD34-positive cells, namely, more angiogenesis. Taken together, hAECs and hAECs-CM may restore ovarian function by regulating expression of VEGFA and its receptors, thus inducing angiogenesis and increasing the follicular growth related to paracrine activity.

So if VEGFA and its receptors played a role in restoring ovarian function, can we just treat POI with administration of VEGFA? Takehara et al. [34] demonstrated that only a small recovery was induced when VEGFA was administered into mouse ovaries. Recently, it has been demonstrated that human hAECs secrete a variety of growth factors, such as epidermal and fibroblast growth factors (HB-EGF, EGF-2, bFGF, FGF-4, FGF-6, and FGF-7), angiogenic growth factors (VEGF, VEGF-D, VEGF-R2, and VEGF-R3), insulin-like growth factors (IGF-1, IGF-ISR, IGFBP-1, and IGFBP-4), and platelet-derived growth factors (PDGF-AA, PDGF-BB, PDGFRa, and PDGFRb) [35]. Therefore, ovaries damaged by chemotherapy needed not only VEGFA for restoration but also other growth factors secreted by hAECs.

## 5. Conclusion

The present study demonstrates that IP injection of hAECs and hAEC-CM could partly restore ovarian function and hAECs-CM seemed sufficient to improve ovarian function of chemotherapy-induced ovarian damage. In addition, our findings suggest that the possible mechanism by which hAECs participate in reviving ovarian function is by regulating VEGFA and its receptors to induce follicular growth related to paracrine activity. Further investigation is needed to determine whether increasing frequency of IP administration of condition medium from hAECs or direct administration of hAECs-CM into the damaged ovaries could more effectively restore ovarian function.

## Supplementary Material

Supplemental Figure 1: Immunohistochemical analysis for VEGFR1 expression in mice ovaries of different groups one month after treatment; Ovarian sections without primary antibody were served as negative controls. (A): control group; (B): Bu/Cy administrated group; (C): Bu/Cy±hAECs(24h) group; (D): Bu/Cy±hAECs-CM(24h) group; (E): Bu/Cy±hAECs(7d) group; (F): Bu/Cy±hAECs-CM(7d) group; (G): negative control. Scale bar: 100 µm.
Supplemental Figure2: Immunohistochemical analysis for VEGFR2 expression in mice ovaries of different groups one month after treatment; Ovarian sections with no primary antibody were served as negative controls. (A): control group; (B): Bu/Cy-treated group; (C): Bu/Cy±hAECs(24h) group; (D): Bu/Cy±hAECs-CM(24h) group; (E): Bu/Cy±hAECs(7d) group; (F): Bu/Cy±hAECs-CM(7d) group; (G): negative control. Scale bar: 100 µm.
Supplemental Figure 3: Immunohistochemical analysis for VEGFA expression in mice ovaries of different groups one month after treatment; Ovarian sections with no primary antibody were served as negative controls. (A): control group; (B): Bu/Cy-treated group; (C): Bu/Cy±hAECs(24h) group; (D): Bu/Cy±hAECs-CM(24h) group; (E): Bu/Cy±hAECs(7d) group; (F): Bu/Cy±hAECs-CM(7d) group; (G): negative control. Scale bar: 100 µm.

## Figures and Tables

**Table 1:** Primers sequence for real-time PCR (hAECs).

Gene	Primer sequence 5′→3′		Amplicon size (bp)
*Oct4 *	Forward	GGCCCGAAAGAGAAAGCGAACC	224
	Reverse	ACCCAGCAGCCTCAAAATCCTCTC	

*Nanog *	Forward	TTCCTTCCTCCATGGATCTG	213
	Reverse	TCTGCTGGAGGCTGAGGTAT	

*SOX2 *	Forward	GCCGAGTGGAAACTTTTGTC	264
	Reverse	GTTCATGTGCGCGTAACTGT	

*E-cadherin *	Forward	TGAGCTTGCGGAAGTCAGTT	219
	Reverse	ACCGTGAACGTGTAGCTCT	

*N-cadherin *	Forward	CGCCATCCGCTCCACTT	227
	Reverse	GGACTCGCACCAGGAGTAAT	

*Vimentin *	Forward	CTCTGGCACGTCTTGACCTT	231
	Reverse	ACCATTCTTCTGCCTCCTGC	

*CK19 *	Forward	CCTACAGCTATCGCCAGTCG	243
	Reverse	TGGTTAGCTTCTCGTTGCCC	

*Snail *	Forward	CTCGGACCTTCTCCCGAATG	223
	Reverse	TCATCAAAGTCCTGTGGGGC	

*Beta-actin *	Forward	TCGCCTTTGCCGATCC	202
	Reverse	GAATCCTTCTGACCCATGCC	

**Table 2:** Primers sequence for real-time PCR (mice ovaries).

Gene	Primer sequence 5′→3′		Amplicon size (bp)
VEGFA	Forward	GCAGCGACAAGGCAGACTAT	169
	Reverse	AACCTCCTCAAACCGTTGGC	

VEGFR1	Forward	TCAAGCTAGAGGTGTCCCCG	152
	Reverse	CTCGGCACCTATAGACACCC	

VEGFR2	Forward	GGCGGTGGTGACAGTATCTT	152
	Reverse	GAGGCGATGAATGGTGATCT	

Beta-actin	Forward	TGGCTCCTAGCACCATGAAG	193
	Reverse	AACGCAGCTCAGTAACAGTCC	
