# Supplementary material for: The Paracrine Effect of Transplanted Human Amniotic Epithelial Cells on Ovarian Function Improvement in a Mouse Model of Chemotherapy-Induced Primary Ovarian Insufficiency
Source: Stem Cells Int. 2015 Nov 9;2016:4148923. doi: 10.1155/2016/4148923 (PMC4655291; doi:10.1155/2016/4148923)

Supplemental Figure 1: Immunohistochemical analysis for VEGFR1 expression in mice ovaries of different groups one month after treatment; Ovarian sections with no primary antibody were served as negative controls. (A): control group; (B): Bu/Cy administrated group; (C): Bu/Cy+hAECs(24h) group; (D): Bu/Cy+hAECs-CM(24h) group; (E): Bu/Cy+hAECs(7d) group; (F): Bu/Cy+hAECs-CM(7d) group; (G): negative control. Scale bar: 100 μm.

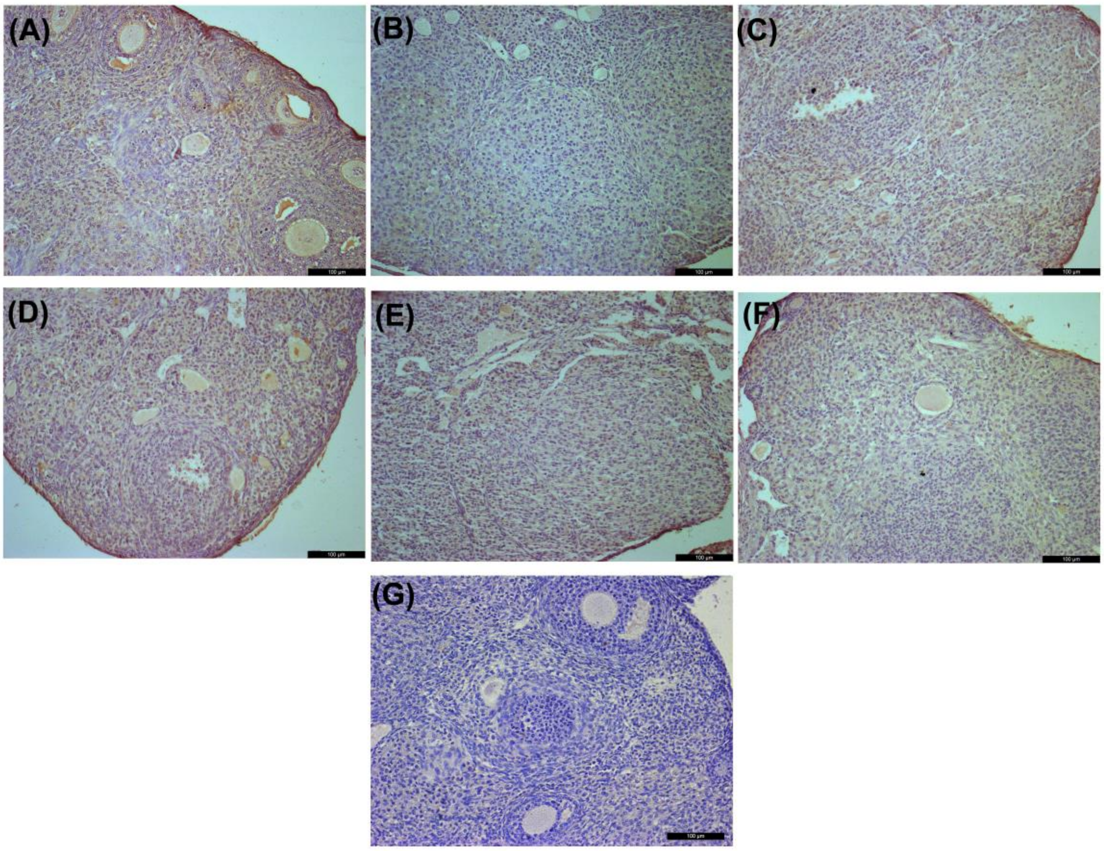

Supplemental Figure2: Immunohistochemical analysis for VEGFR2 expression in mice ovaries of different groups one month after treatment; Ovarian sections with no primary antibody were served as negative controls. (A): control group; (B): Bu/Cy-treated group; (C): Bu/Cy+hAECs(24h) group; (D): Bu/Cy+hAECs-CM(24h) group; (E): Bu/Cy+hAECs(7d) group; (F): Bu/Cy+hAECs-CM(7d) group; (G): negative control. Scale bar: 100 μm.

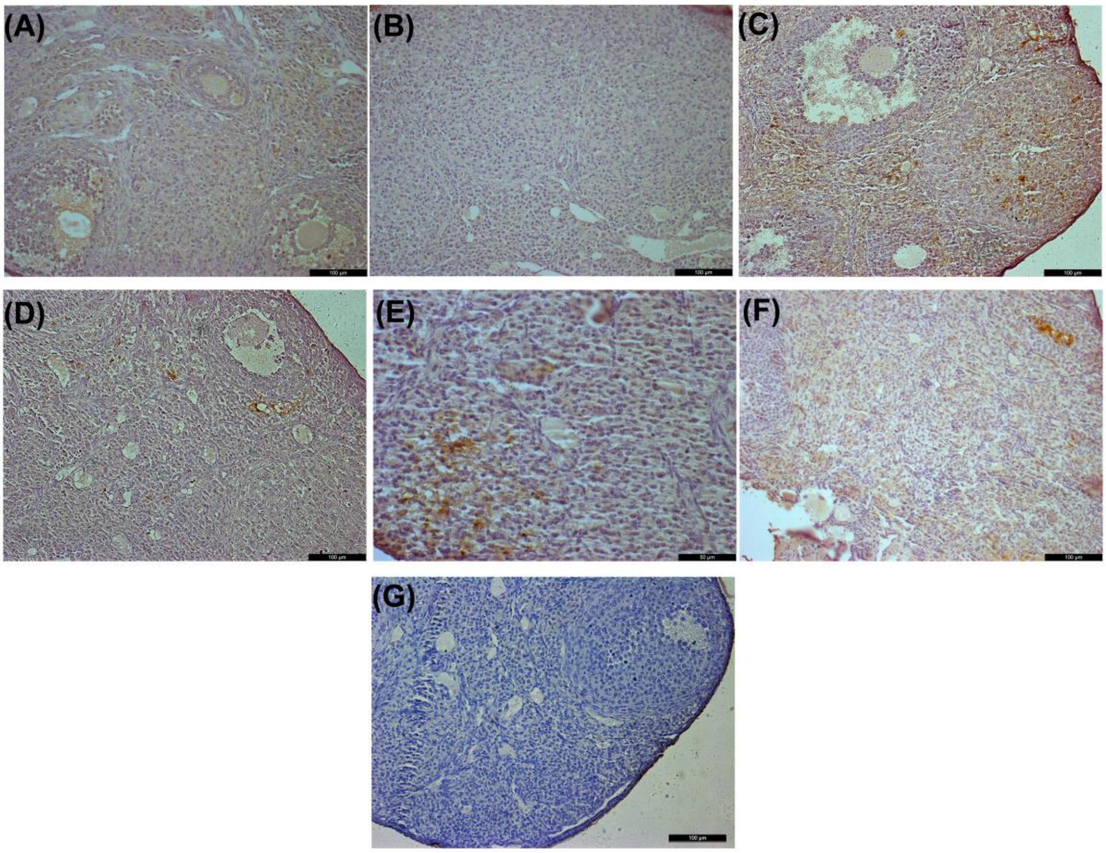

Supplemental Figure 3: Immunohistochemical analysis for VEGFA expression in mice ovaries of different groups one month after treatment; Ovarian sections with no primary antibody were served as negative controls. (A): control group; (B): Bu/Cy-treated group; (C): Bu/Cy+hAECs(24h) group; (D): Bu/Cy+hAECs-CM(24h) group; (E): Bu/Cy+hAECs(7d) group; (F): Bu/Cy+hAECs-CM(7d) group; (G): negative control. Scale bar: 100 μm.

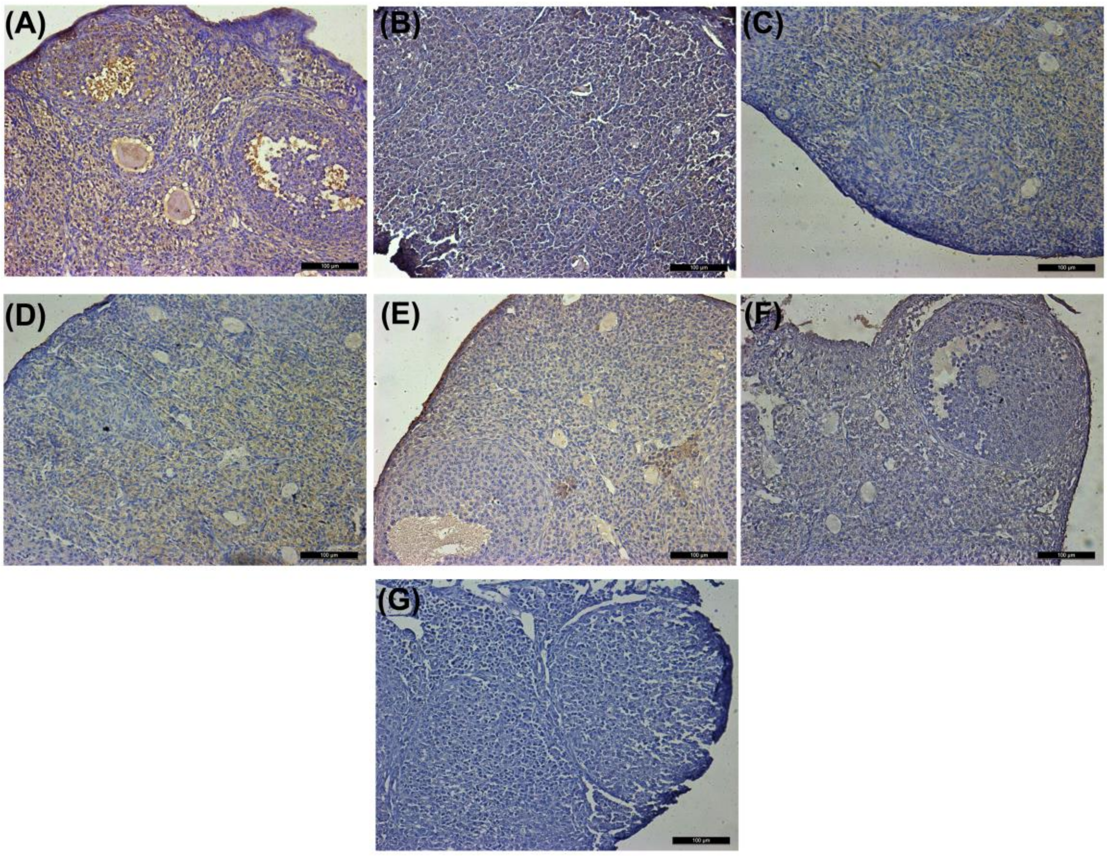

Supplement: Supplementary file 1 — Supplemental Figure 1: Immunohistochemical analysis for VEGFR1 expression in mice ovaries of different groups one month after treatment; Ovarian sections without primary antibody were served as negative controls. (A): control group; (B): Bu/Cy administrated group; (C): Bu/Cy±hAECs(24h) group; (D): Bu/Cy±hAECs-CM(24h) group; (E): Bu/Cy±hAECs(7d) group; (F): Bu/Cy±hAECs-CM(7d) group; (G): negative control. Scale bar: 100 µm. Supplemental Figure2: Immunohistochemical analysis for VEGFR2 expression in mice ovaries of different groups one month after treatment; Ovarian sections with no primary antibody were served as negative controls. (A): control group; (B): Bu/Cy-treated group; (C): Bu/Cy±hAECs(24h) group; (D): Bu/Cy±hAECs-CM(24h) group; (E): Bu/Cy±hAECs(7d) group; (F): Bu/Cy±hAECs-CM(7d) group; (G): negative control. Scale bar: 100 µm. Supplemental Figure 3: Immunohistochemical analysis for VEGFA expression in mice ovaries of different groups one month after treatment; Ovarian sections with no primary antibody were served as negative controls. (A): control group; (B): Bu/Cy-treated group; (C): Bu/Cy±hAECs(24h) group; (D): Bu/Cy±hAECs-CM(24h) group; (E): Bu/Cy±hAECs(7d) group; (F): Bu/Cy±hAECs-CM(7d) group; (G): negative control. Scale bar: 100 µm. [file 4148923.f1.pdf]
